# Ultrastructural patterns of the excretory ducts of basal neodermatan groups (Platyhelminthes) and new protonephridial characters of basal cestodes

**DOI:** 10.1186/s13071-020-04307-8

**Published:** 2020-09-04

**Authors:** Larisa G. Poddubnaya, Roman Kuchta, Tomáš Scholz

**Affiliations:** 1 Institute for Biology of Inland Waters, Russian Academy of Sciences, Borok, Yaroslavl Region 152742 Russia; 2 Institute of Parasitology, Biology Centre, Biology Centre of the Czech Academy of Sciences, České Budějovice, Czech Republic

**Keywords:** Neodermata, Excretory system, Ultrastructure, TEM, Phylogeny, Cestoda, Trematoda, Monogenea

## Abstract

**Background:**

The flatworms (Lophotrochozoa: Platyhelminthes) are one of the major phyla of invertebrates but their interrelationships are still not well understood including unravelling the most closely related taxon of the Neodermata, which includes exclusively obligate parasites of all main groups of vertebrates with some 60,000 estimated species. Recent phylogenomic studies indicate that the freshwater ‘microturbellarian’ *Bothrioplana semperi* may be the closest ancestor to the Neodermata, but this hypothesis receives little morphological support. Therefore, additional morphological and ultrastructural characters that might help understand interrelations within the Neodermata are needed.

**Methods:**

Ultrastructure of the excretory ducts of representatives of the most basal parasitic flatworms (Neodermata), namely monocotylid (Monopisthocotylea) and chimaericolid (Polyopisthocotylea) monogeneans, aspidogastreans (Trematoda), as well as gyrocotylidean and amphilinidean tapeworms (Cestoda), were studied using transmission electron microscopy (TEM).

**Results:**

The present study revealed the same pattern of the cytoarchitecture of excretory ducts in all studied species of the basal neodermatans. This pattern is characterised by the presence of septate junctions between the adjacent epithelial cells and lateral ciliary flames along different levels of the excretory ducts. Additionally, a new character was observed in the protonephridial terminal cell of *Gyrocotyle urna*, namely a septate junction between terminal and adjacent duct cells at the level of the distal extremity of the flame tuft. In *Amphilina foliacea*, a new type of protonephridial cell with multiple flame bulbs and unique character of its weir, which consists of a single row of the ribs, is described. A remarkable difference has been observed between the structure of the luminal surface of the excretory ducts of the studied basal neodermatan groups and *B. semperi*.

**Conclusions:**

The present study does not provide ultrastructural support for a close relationship between the Neodermata and *B. semperi*.
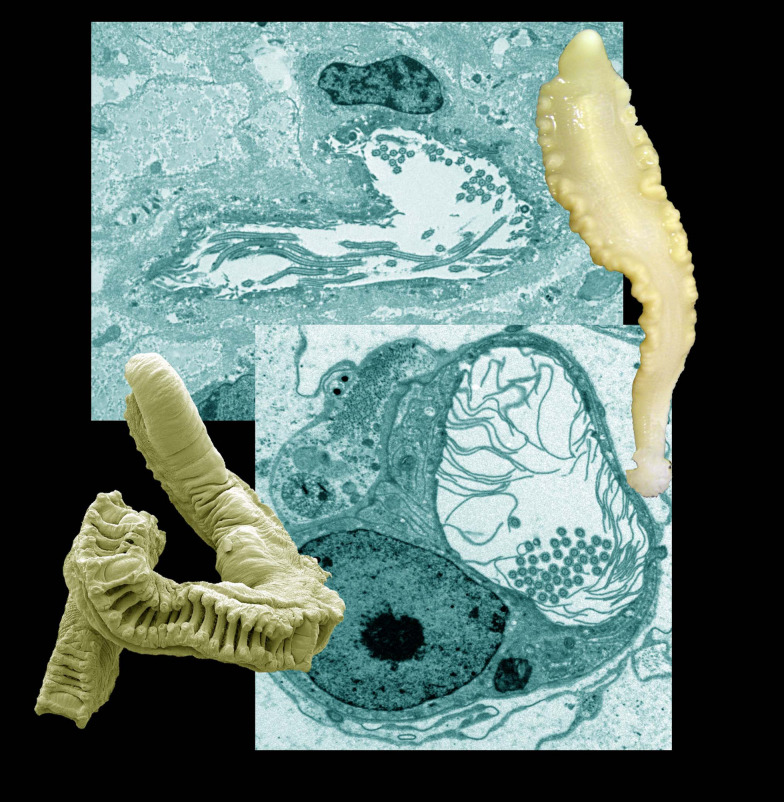

## Background

The flatworms (Lophotrochozoa: Platyhelminthes) are one of the major phyla of animals, with numerous serious pathogens of humans and domestic animals, but their interrelationships are still not well understood [1]. Knowledge of the phylogeny of flatworms has been considerably enriched recently by transcriptomic and genomic data [2, 3]. Although nearly all flatworm lineages contain some symbiotic representatives, Neodermata includes exclusively obligate parasites of all main groups of vertebrates comprising some 60,000 estimated species [1, 4].

However, the most closely related ancestor to the Neodermata remains unknown. Recent phylogenomic studies of Egger et al. [2] and Laumer et al. [3] revealed the inconspicuous freshwater ‘microturbellarian’ *Bothrioplana semperi* Braun, 1881 (Rhabditophora: Bothrioplanida) with cosmopolitan distribution as the putative closest ancestor to all parasitic Neodermata. These authors have also shown that *Bothrioplana* is closely related to either the Proseriata [5] or the Tricladida [2, 3]. However, conclusions by Laumer & Giribet [5], Egger et al. [2] and Laumer et al. [3] based on molecular data have received little morphological support to date [6, 7].

Despite considerable progress in unravelling phylogenetic relationships of platyhelminths over the last decades [1], the interrelationships of the four main lineages of the Neodermata are still not resolved using classical genetic markers (rRNA-based analyses) or mitochondrial genomes [8, 9]. Any of four potential evolutionary scenarios proposed by Littlewood & Waeschenbach [10] may be valid. Therefore, search for additional morphological and ultrastructural characters that might help understand interrelations within the Neodermata and their relationships to the presumably most closely related ancestor such as *B. semperi* is still needed.

The ultrastructure of the protonephridial terminal complex is considered to be useful for the assessment of phylogenetic relationships among platyhelminths [11–14]. However, other structures associated with the protonephridial system such as excretory ducts can also be used to characterise different flatworm groups [13]. Despite the existence of data on the ultrastructure of the excretory ducts of both free-living and parasitic flatworms from different groups, the number of such studies has been low or, in the case of the most basal neodermatan groups, absent [13].

The present study provides data based on ultrastructural study of the excretory ducts of representatives of all four basal lineages of the Neodermata, namely the monopisthocotylean *Calicotyle affinis* Scott, 1911 (Monocotylidae), the polyopisthocotylean *Chimaericola leptogaster* (Leuckart, 1830) (Chimaericolidae), aspidogastreans *Aspidogaster limacoides* Diesing, 1835 (Aspidogastridae) and *Multicalyx elegans* (Olsson, 1869) (Multicalycidae), and the basal cestodes *Gyrocotyle urna* (Grube et Wagener, 1852) (Gyrocotylidea) and *Amphilina foliacea* (Rudolphi, 1819) (Amphilinidea). New data may help in the reconstruction of the origin and diversification of parasitic flatworms and evolution of parasitism. Additionally, the present study includes the first description of the unique protonephridial terminal cell of *A. foliacea* and adds new details about the terminal protonephridial organ of *G. urna*.

## Methods

Adult specimens of *C. leptogaster*, *C. affinis*, *M. elegans* and *G. urna* were recovered from the gills, cloaca, gall-bladder and spiral intestine, respectively, of the rabbit fish, *Chimaera monstrosa* Linnaeus (Chimaeriformes: Chimaeridae), captured from the Norwegian Sea off Tromsø at depths of 500–750 m in June and October 2014, 2015 and 2017. Adult specimens of *A. limacoides* were found in the intestine of the white bream, *Blicca bjoerkna* (Linnaeus) (Cypriniformes: Cyprinidae), from the Rybinsk Reservoir, Russia in May 2018. *Amphilina foliacea* was obtained from the body cavity of the starlet sturgeon, *Acipenser ruthenus* Linnaeus (Acipenseriformes: Acipenseridae), in the Cheboksary Reservoir at the Volga River, Russia in August 2014. Live parasites were fixed in cold 3% glutaraldehyde in 0.1 M sodium cacodylate buffer (pH 7.2) for 20 days at 5 °C, rinsed 4 times for 20 min in the same buffer and post-fixed in 1% osmium tetroxide for 1 h. For ultrastructural (TEM) studies, samples were dehydrated in a graded series of ethanol and acetone and embedded in a mixture of Araldite and Epon. Ultrathin sections (50–90 nm in thickness) were stained with uranyl acetate and lead citrate, and then examined using a JEOL-JEM-1011 transmission electron microscope (TEM) operating at 80 kV (Institute for Biology of Inland Waters, Borok, Russia) and JEOL-JEM-1010 TEM (Institute of Parasitology, Biology Centre, CAS, České Budějovice, Czech Republic).

## Results

### Polyopisthocotylea

#### *Chimaericola leptogaster (Chimaericolidae)*

**Fig. 1:**
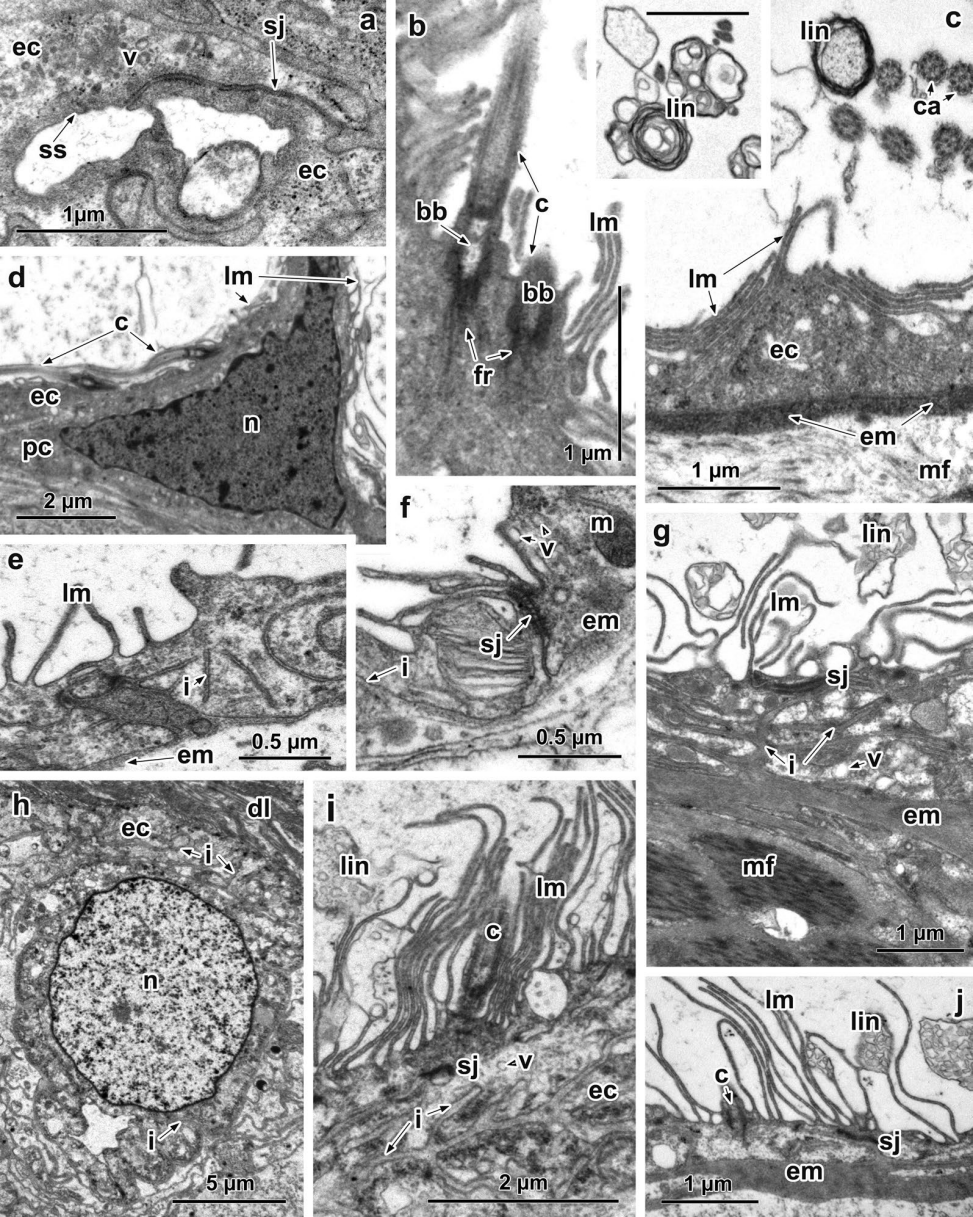
Ultrastructure of the excretory ducts of *Chimaericola leptogaster* (**a**–**f**) and *Calicotyle affinis* (**g**–**j**). **a** Small excretory duct at a short distance from terminal cell. **b** Epithelial lining of the collecting duct; note surface lamellae and cilia of lateral flame. **c** Epithelial wall of the main excretory duct showing inclusions within its lumen; note a thick layer of extracellular matrix supporting epithelial cytoplasm. Insert: luminal inclusions (*scale-bar*: 1 µm). **d** Collecting duct; note basally located nucleus. **e**, **f** Epithelial wall of the collecting ducts showing invaginations of the basal membrane and septate junction. **g** Epithelial cytoplasm of the terminal portion of the main excretory duct showing wide invaginations of the basal membrane filled with extracellular matrix; note thick layer of extracellular matrix and muscle fibres around it. **h** Basally located nucleus of the collecting duct; note invaginations of the plasma membrane into perinuclear cytoplasm. **j** Epithelial wall of the main excretory duct; note surface lamellae, cilium of lateral flame, thick layer of the extracellular matrix. *Abbreviations*: bb, basal body of the cilium; c, cilium; ca, cilium axoneme; dl, duct lumen; ec, epithelial cytoplasm; em, extracellular matrix; fr, fibrous rootlet; i, invaginations of the basal membrane; lin, luminal inclusions; lm, lamellae; m, mitochondrion; mf, muscle fibres; n, nucleus; pc, perinuclear cytoplasm; sj, septate junction; ss, smooth luminal surface; v, vesicles

A short distance beyond the tip of cilia of the terminal cell, the narrow epithelial lining of the proximal duct cell is smooth; small vesicles with a variable electron density are present within the epithelial cytoplasm (Fig. 1a). Long septate junctions are present linking adjacent cells (Fig. 1a). At some distance from the terminal cell, the luminal surfaces of the excretory collecting ducts bear lamellae (Fig. 1b–f), and bundles of cilia forming the lateral ciliary tufts extend into the duct lumen where they are common along its entire length, although there is no pattern in the arrangement of these bundles (Fig. 1b, d). The cilia in each lateral ciliary tuft are not numerous and only 2 cilia were observed in TEM sections 50–90 nm thick (Fig. 1b, d). Each cilium has a cylindrical basal body with a short, straight fibrous rootlet, which is embedded in the epithelial lining of the excretory ducts (Fig. 1b, d). Each excretory epithelial cell contains a large, basally located nucleus, perinuclear cytoplasm with free ribosomes, mitochondria and small electron-lucent vesicles (Fig. 1d). A small number of invaginations of the basal plasma membrane of the collecting ducts extend into the epithelial cytoplasm (Fig. 1e, f). A thin layer of extracellular basal matrix is beneath the basal plasma membrane (Fig. 1e, f). The epithelial lining of the excretory ducts lacks hemidesmosomes connecting it to the underlying extracellular matrix. Adjacent cells are connected by septate junctions in apically located sites (Fig. 1f). The epithelial lining of the main excretory ducts is supported by a layer of thick, densely packed extracellular matrix and muscle fibres (Fig. 1c). The luminal surfaces of the main ducts bear lamellae and lateral ciliary tufts; membrane-bound inclusions of varied shape and content are apparent within the duct lumen (Fig. 1c, insert).

### Monopisthocotylea

#### *Calicotyle affinis (Monocotylidae)*

As in *C. leptogaster*, the luminal surface of the narrow excretory duct is smooth, distal to the tip of each ciliary tuft of the terminal cell. Further from the terminal flame, the luminal surface of the collecting ducts forms numerous thin lamellae (Fig. 1i). A characteristic feature of both the epithelial lining and perinuclear cytoplasm of the basally located nucleus of each excretory cell is the presence in their cytoplasm of numerous, deep, wide, branching invaginations of the basal plasma membrane supported by extracellular matrix (Fig. 1g–i). Between these wide invaginations, the epithelial cytoplasmic matrix is relatively translucent, with local concentrations of free ribosomes, mitochondria and electron-lucent vesicles of different size (Fig. 1g, i, j). Septate junctions occur in the walls of all excretory collecting ducts (Fig. 1g, j). Throughout the lengths of the different collecting ducts, including the main ducts, single cilia can frequently be seen or more rarely three cilia are apparent in TEM sections 50–90 nm thick (Fig. 1i, j). Every cilium has a distinct basal body with a short straight, fibrous rootlet (Fig. 1j). The terminal parts of the main excretory ducts possess well-developed underlying muscle layers (Fig. 1g). Membrane-bound inclusions are observed in the lumen of collecting and main ducts (Fig. 1g, i, j).

### Trematoda: Aspidogastrea

#### *Aspidogaster limacoides (Aspidogastridae)*

**Fig. 2:**
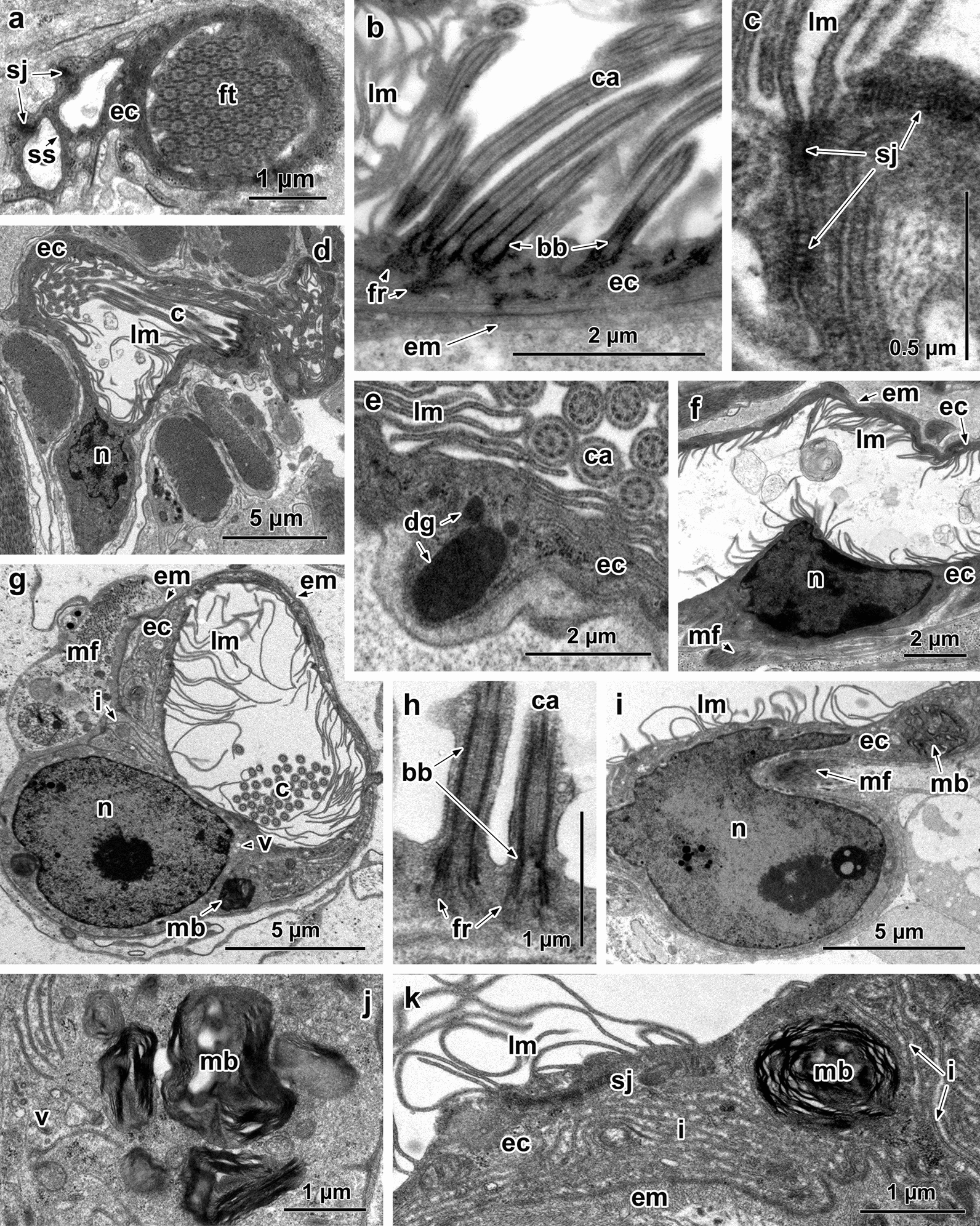
Ultrastructure of the excretory ducts of *Aspidogaster limacoides* (**a**–**f**) and *Multicalyx elegans* (**g**–**k**) **a** Epithelial wall of the adjacent proximal duct cell; note smooth luminal surface and septate junctions. **b** Epithelial lining of the collecting duct showing lateral ciliary flame. **c** Septate junction in the epithelial cytoplasm. **d** Basally located nucleus of the epithelial cell; note lamellae and lateral flame on the luminal surface. **e** Electron-dense granules in the epithelial cytoplasm of the collecting duct. **f** Apical position of the nucleus of the main collecting duct. **g** Lamellae and lateral flame on the luminal surface. **h** Long basal body and weakly define rootlet fibres of lateral flame cilia. **i, k** Epithelial lining of the main excretory duct; note nucleus, myelin-like bodies, septate junction and deep basal invaginations. **j** Collecting excretory duct with basal nucleus and myelin-like bodies in the perinuclear cytoplasm. *Abbreviations*: bb, basal body of the cilium; c, cilium; ca, cilium axoneme; dg, dense granules; ec, epithelial cytoplasm; em, extracellular matrix; fr, fibrous rootlet; ft, flame tuft; i, invaginations of the basal membrane; lm, lamellae; mb, myelin-like bodies; mf, muscle fibres; n, nucleus; sj, septate junction; ss, smooth luminal surface; v, vesicles

The epithelial lining of the first canal cell has smooth luminal surface and septate junctions extending between its basal and luminal membranes (Fig. 2a). Cytoplasm of the epithelial cells is homogeneously electron-dense and contains large numbers of free ribosomes (Fig. 2a). The luminal surface of the collecting ducts is elevated into long, thin lamellae, and the cilia of the lateral flames arise from this surface and extend into the duct lumen (Fig. 2b, d). In longitudinal sections of 50–90 nm in thickness the number of cilia ranges from 5 to 7 (Fig. 2b, d). Each ciliary axoneme arises from a basal body and straight rootlet fibres (Fig. 2b). The epithelial cytoplasm of the excretory ducts is marked by numerous free ribosomes and electron-dense granules of different sizes (Fig. 2e). Septate junctions between adjacent cells are present in all excretory ducts and may form a long border zone (Fig. 2c). The epithelium of the ducts lies on a thin extracellular matrix with sparse circular muscle fibrils beneath (Fig. 2b, f). Nuclei in the epithelial wall of the collecting ducts are located close to the basal membrane (Fig. 2d) unlike those in the main collecting ducts where the nuclei occupy a more apical position (Fig. 2f). The luminal surface of the main excretory ducts is enlarged by lamellae, but lateral flames were not observed (Fig. 2f).

#### *Multicalyx elegans (Multicalycidae)*

As in *A. limacoides*, the luminal surface of epithelial cells forming the wall of the excretory collecting ducts of *M. elegans* is distinguished by the presence of lamellae and lateral flames (Fig. 2g). The nucleus of each epithelial cell of both the collecting and main excretory ducts is located basally (Fig. 2g, i). Both perinuclear and general cell cytoplasm contain, in addition to free ribosomes, small electron-lucent vesicles and large bodies with a myelin-like electron-dense content (Fig. 2g, i, j). In each longitudinal section there are 4–5 cilia; they are characterised by a long basal body and weakly defined fibres of the rootlet (Fig. 2h). The main excretory ducts lack lateral flames (Fig. 2i, k). Septate junctions extend along the epithelial wall of all excretory ducts, and the basal cell membranes of these ducts form deep invaginations into the epithelial wall (Fig. 2g, k). A thin layer of extracellular matrix surrounds the epithelial wall of all excretory ducts (Fig. 2g, k).

### Cestoda

#### *Gyrocotyle urna (Gyrocotylidea)*

**Fig. 3:**
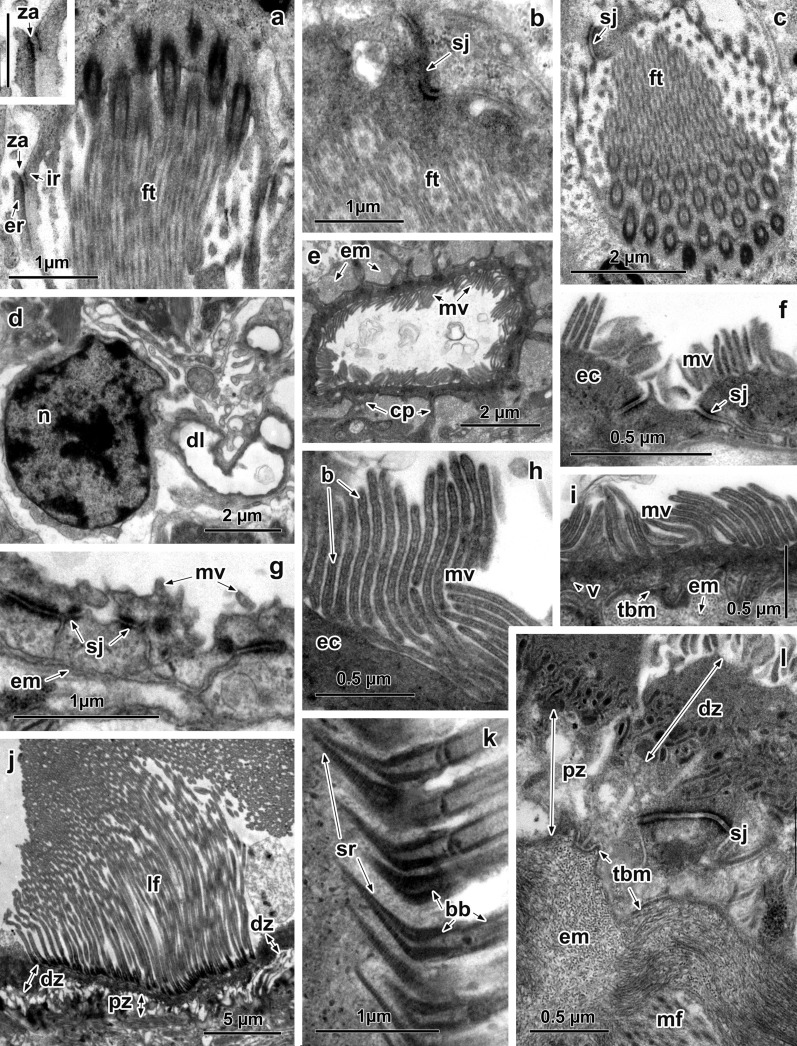
Ultrastructure of the protonephridial complex and excretory ducts of *Gyrocotyle urna*. **a** Longitudinal section through the flame tuft; note *zonulae adherentes* between the internal and external ribs (insert: *scale-bar*: 0.5 µm). **b** Oblique section through distal extremity of the flame tuft; note septate junction between neighbouring terminal and proximal cells. **c** Terminal flame tuft showing septate junction at the distal extremity. **d** Adjacent proximal duct cell. **e**, **f** Larger excretory duct; note numerous surface microvilli, cytoplasmic processes and septate junction. **g** Epithelial wall of small excretory duct; note short surface microvilli and septate junction. **h** Luminal surface with microvilli; note thin electron-dense band in its central area. **i** Epithelial wall of larger excretory duct; note basal extracellular matrix supporting basal membrane. **j**, **l** Epithelial wall of the largest excretory duct showing large lateral flame, two distinct zones of the epithelial cytoplasm, septate junction and thick layer of extracellular matrix. **k** Inserted striated conical rootlets of the cilia of the lateral flame. *Abbreviations*: b, medial band of microvilli; bb, basal body of the cilium; cp, cytoplasmic process; dl, duct lumen; ec, epithelial cytoplasm; em, extracellular matrix; er, external rib; fr, fibrous rootlet; ft, flame tuft; i, invaginations of the basal membrane; ir, internal rib; lf, lateral flame; mf, muscle fibres; mv, microvilli; n, nucleus; sj, septate junction; sr, striated rootlet; v, vesicles; za, *zonula adherens*

A series of longitudinal sections through the terminal protonephridial flame of *G. urna* has shown that, between the internal ribs (arising from the terminal cell) and external ribs (extending from the proximal canal cell), the adjoining membranes are lined by electron-dense material which forms contact sites that can be identified as *zonulae adherentes* (Fig. 3a, insert). Moreover, septate junctions can be seen between neighbouring terminal and proximal cells of the terminal organ in sections at the level of the distal extremity of the flame tuft (Fig. 3b, c).

The cell body of the proximal duct cell is located underneath the basal membrane and has a large, ovoid nucleus with dense patches of peripheral and central chromatin (Fig. 3d). Both the narrow area of perinuclear cytoplasm and the duct epithelium have a homogeneous, electron-dense content with free ribosomes and small, electron-lucent vesicles (Fig. 3b, d). The epithelial wall of the small excretory ducts is characterised by infrequent, short microvilli (~ 0.2 µm in length) on its luminal surface and the presence of septate junctions intersecting the epithelial lining (Fig. 3g). A thin layer of basal extracellular matrix but no muscle fibres are present beneath the basal membrane of the epithelial cells forming these ducts (Fig. 3g).

The luminal surface of the larger excretory ducts is increased by numerous microvilli ~ 1 µm long and with a thin, medial, electron-dense band (Fig. 3e, f, h, i). Epithelial nuclei occur at some distance from the epithelial lining and are joined to this lining by narrow cytoplasmic processes; septate junctions penetrate the epithelial cytoplasm (Fig. 3e, f). The dense epithelial cytoplasm contains numerous free ribosomes and small vesicles are concentrated in the basal region of the duct wall epithelium (Fig. 3i). The basal plasma membrane of the duct epithelial cells is usually supported by a thin, tightly packed basal matrix, below which is a thicker layer of loosely packed extracellular matrix; muscle fibres are scattered irregularly around these ducts (Fig. 3e, i).

In addition to surface microvilli and septate junctions, in the epithelial cytoplasm of the larger excretory ducts there are large lateral ciliary tufts projecting into the duct lumen (Fig. 3j). Such ciliary tufts can include up to 50 cilia. Each cilium is ~ 12 µm long and is inserted into the duct epithelium, where there is a long basal body and well-developed, striated conical, bent rootlets (Fig. 3k). The epithelial cytoplasm of these ducts contains two distinct zones, differing in the density of their cytoplasm (Fig. 3j, l). A dense cytoplasmic zone is localised immediately beneath the surface plasma membrane; it is filled with free ribosomes and a thin layer of electron-dense bodies. Small vesicles are apparent within this zone (Fig. 3j-l). The second zone is a pale cytoplasmic area filled with electron-lucent vesicles of different sizes and shapes (Fig. 3j, l). There is a thin layer of tightly packed basal matrix, beneath which is a wide layer of loosely packed extracellular matrix separating the epithelial duct wall from the underlying muscles (Fig. 3l).

#### *Amphilina foliacea (Amphilinidea)*

**Fig. 4:**
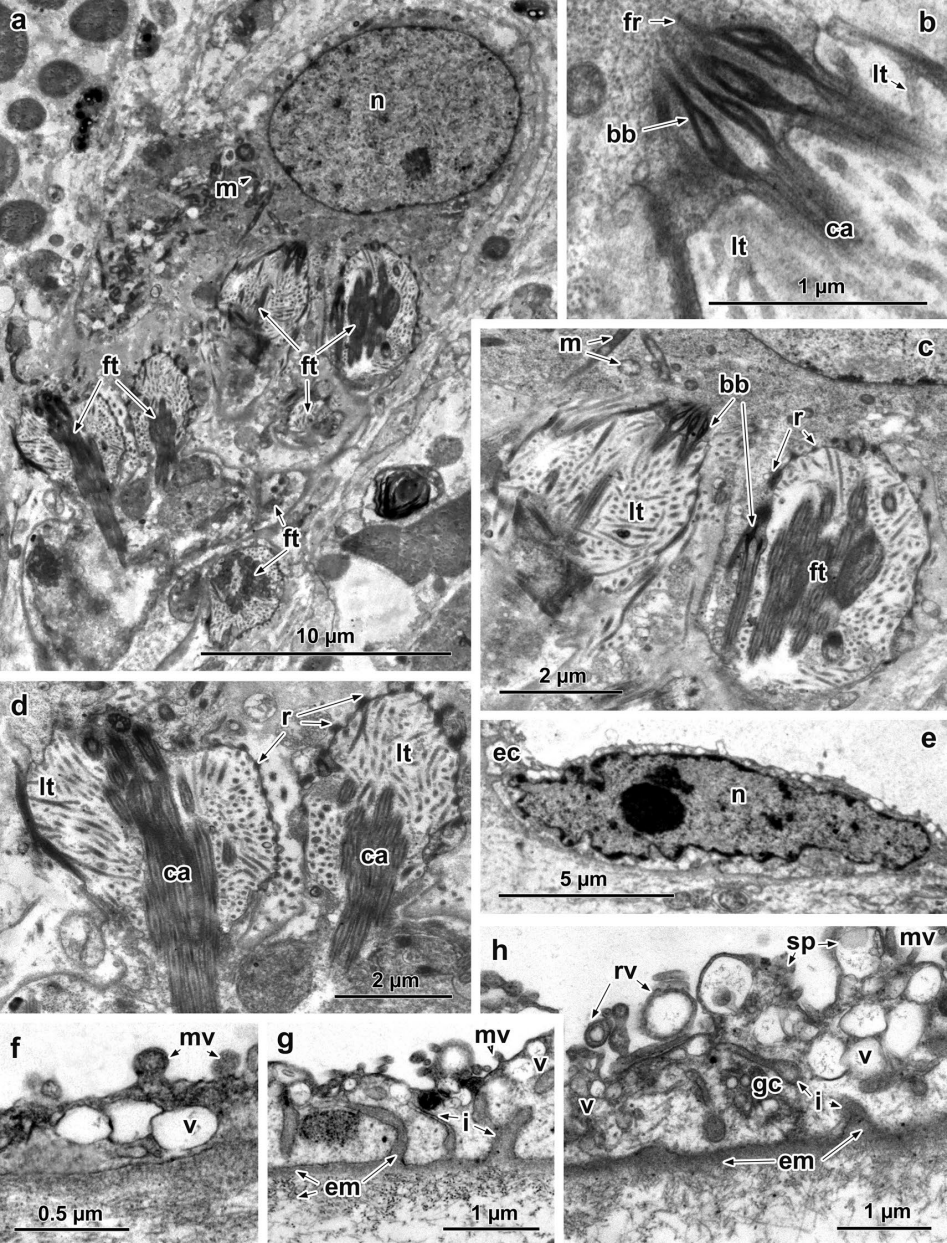
Ultrastructure of the special terminal cell and excretory ducts of *Amphilina foliacea*. **a** Terminal cell with multiple flames of different directions. **b** Anchored portion of flame cilia into terminal cell cytoplasm, note expanded lateral edges of the basal bodies. **c** Two flame bulbs within terminal cell. **d** Flame bulbs showing weir with one row of dense ribs joined thin dense sheath and numerous leptotriches filled the space of the bulbs. **e** Nucleus of the excretory duct. **f** Small excretory duct, note surface microvilli. **g**, **h** Large excretory ducts showing large basal invaginations filled with extracellular matrix. *Abbreviations*: bb, basal body of the cilium; ca, cilium axoneme; ec, epithelial cytoplasm; em, extracellular matrix; fr, fibrous rootlet; ft, flame tuft; gc, Golgi complex; i, invaginations of the basal membrane; lt, leptotriches; m, mitochondrion; mv, microvilli; n, nucleus; r, rib; rv, released vesicles; sp, surface processes; v, vesicles

Along with the typical terminal organ, i.e. a combination of 2 cells, a terminal cell bears the tuft of cilia and the first canal cell; another unusual type of terminal cell is present in adult *A. foliacea* (Fig. 4a). In a longitudinal section of this terminal cell, 6 ciliary tufts (~ 5 µm long) are inserted in the cytoplasm (Fig. 4a). These tufts are dispersed in different directions within the cell cytoplasm (Fig. 4a). There are 3–4 cilia across the diameter of each tuft (Fig. 4b–d). Each tuft cilium is anchored to the cytoplasm of the terminal cell by a well-developed basal body that, in longitudinal section, possesses expanded, elongated, electron-dense lateral edges and short processes of straight rootlet fibres (Fig. 4d). The weir (filtration apparatus) of each tuft comprises one row of ribs joined by a thin, electron-dense sheath (Fig. 4d). The ribs bear numerous, long inner leptotriches, which fill the space between the tuft and the weir (Fig. 4c, d).

All small and large excretory ducts in this species lack septate junctions. The luminal surface of the small ducts is enlarged by short, mushroom-shaped microvilli about 0.1–0.3 µm long (Fig. 4f). Their epithelial cytoplasm contains numerous free ribosomes and round, electron-lucent vesicles, and a thin layer of extracellular matrix is present beneath the basal plasma membrane (Fig. 4f). The luminal surface of the large excretory ducts bears surface processes, differing in size and shape, on which short microvilli can be seen occasionally (Fig. 4g, h). Electron-lucent vesicles of various sizes are present in the epithelial wall and around Golgi complexes (Fig. 4g, h). These vesicles occur in close proximity to the luminal surface, with which they unite, resulting in the release of their contents into the duct lumen (Fig. 4g, h). The basal membrane has large invaginations, which penetrate deeply into the epithelial wall; such invaginations are filled with the extracellular matrix, which surrounds the duct wall (Fig. 4g). In all excretory ducts, only large, intraepithelial nuclei are apparent (Fig. 4e).

## Discussion

### Structural patterns of the excretory ducts of the basal neodermatan groups

In the present study we compared the cytoarchitecture of the excretory ducts of the species belonging to the basal neodermatan groups. Monocotylid and chimaericolid monogeneans are considered to be close to the base of the monopisthocotylean and polyopisthocotylean lineages, respectively [15–18]. The Aspidogastrea (Trematoda) is the sister group of the Digenea [19–21] and the Gyrocotylidea and Amphilinidea are the most basal groups of the Cestoda [22–25]. The data previously available on the ultrastructure of the excretory duct wall of neodermatans indicated that the flattened epithelium of the excretory ducts possesses nuclei located distally or proximally to the basal membrane and that adjacent cells are connected or not by septate junctions [13].

**Fig. 5:**
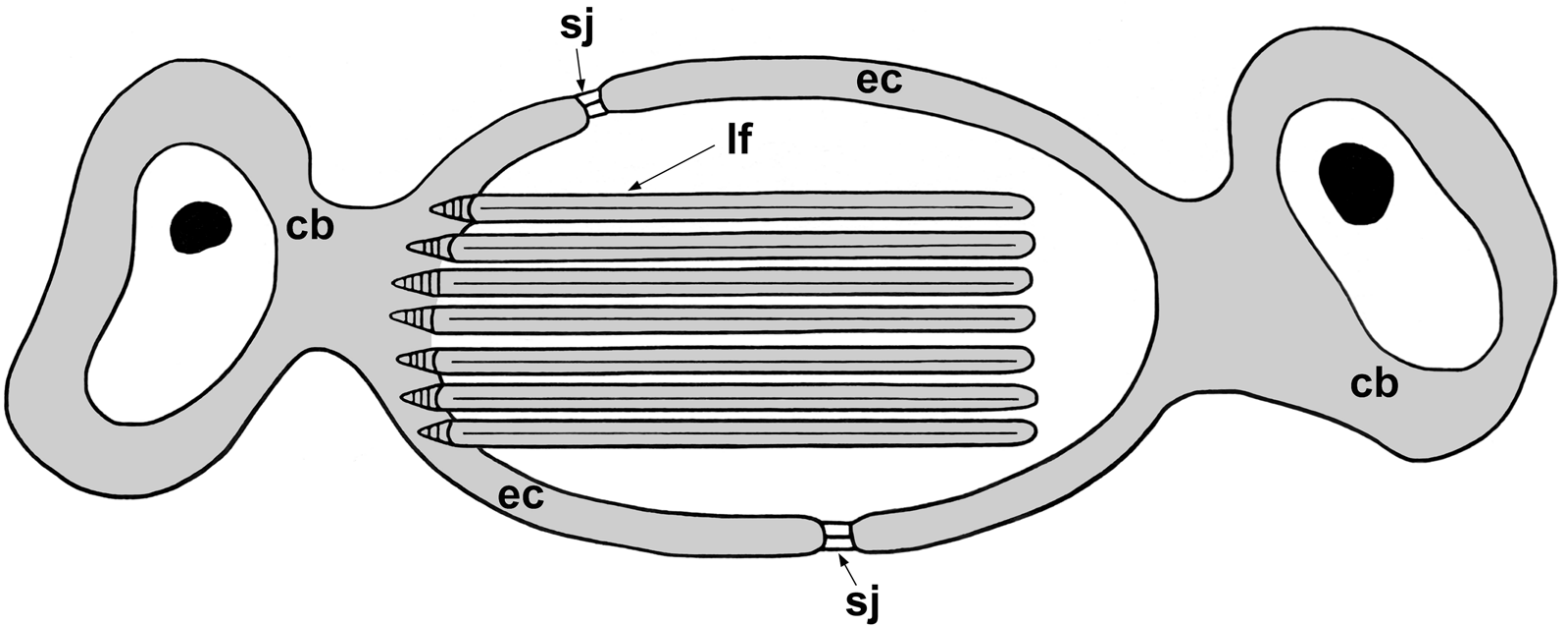
Pattern of the excretory ducts of basal neodermatans: Chimaericolidae, Monocotylidae, Aspidogastridea and Gyrocotylidea. *Abbreviations*: cb, cell body; ec, epithelial cytoplasm; lf, lateral flame; sj, septate junction

Our investigation has shown that all studied species except for *A. foliacea* have the same pattern in the cytoarchitecture of their excretory ducts. This pattern is characterised by the presence of septate junctions between adjacent epithelial cells and lateral ciliary tufts in the excretory ducts of the different levels (Fig. 5). Previous observations of three other species of aspidogastrids, namely *Multicotyle purvisi*, *Lobatostoma manteri* and *Rugogaster hydrolagi*, showed the same pattern of their excretory ducts [26–28]. Concerning ultrastructural data on the excretory ducts of gyrocotylideans, the presence of the septate junctions in the epithelial wall of excretory ducts of *G. urna* has not been found previously [29]. In contrast to previously studied monogeneans and aspidogastreans which possess lateral ciliary tufts along different levels of the excretory ducts, lateral ciliary tufts have only been observed in the larger excretory ducts of *G. urna* ([29]; present study). Similarly, as in the basal neodermatan groups, the presence of septate junctions in the excretory duct wall is a character of the most free-living flatworms [13]. The presence of lateral ciliary tufts has also been observed in the excretory ducts of free-living Tricladida (*Artioposthia*, *Bdellocephala*, *Dugesia*, *Romankenkuis*), Proseriata (*Invenusta*, *Mococelis*) and Rhabdocoela (*Actinodactylella*, *Rhinolasius, Gieysztoria*), but the species of the last group do not possess septate junctions in the excretory ducts [13, 30–33]. However, the excretory ducts of the presumably most closely related *Bothrioplana* lack lateral flames [6]. It should be noted that the absence of the septate junctions and lateral ciliary tufts are characteristic traits of amphilinideans ([25, 29, 34]; present study]) and the Eucestoda [25, 35].

**Fig. 6:**
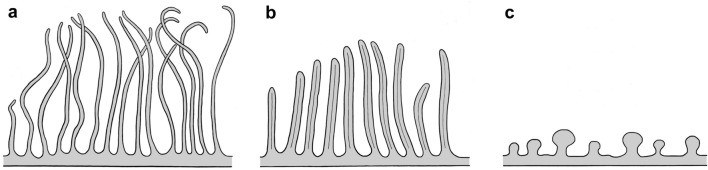
Luminal surface structures of the excretory ducts of basal neodermatans. **a** Lamellae of the basal monogeneans and aspidogastreans. **b** Microvilli of gyrocotylideans. **c** Microvilli of amphilinideans

A remarkable difference between the studied basal neodermatan groups (monopisthocotylean and polypisthocotylean monogeneans and aspidogastreans) and *Bothrioplana semperi* appears to be in the structure of the luminal surface of the excretory ducts (Fig. 6). In the above-mentioned neodermatan groups the duct surface bears lamellae, whereas in *B. semperi*, which is assumed to be neodermatan ancestor, the excretory duct epithelium is enlarged with “rare rounded short microvilli” and there are longer microvilli on the luminal surface in larger ducts [6]. Unfortunately, Kornakova [6] did not provide any detailed illustration to better visualise her description of the excretory ducts of *B. semperi*. Among free-living flatworms, microvilli have been recorded in large excretory ducts of the rhabdocoelid *Gieysztoria* sp. [32]. Lamellae as luminal structures of the excretory ducts are characteristic of proseriates *Monocelis* sp. and *Invenusta paracnida* [30, 33]. Among basal neodermatans, the enlargement of the luminal surface of the excretory ducts by microvilli is observed in larval and adult gyrocotylid *G. urna* ([29, 36]; present study), the amphilinid *A. foliacea* ([29]; present study) and *A. elongata* [34], and in all members of the Eucestoda [35]. Interestingly, as shown by our investigation, slender microvilli with the central narrow electron-dense band along its whole length have been observed on the luminal surface of the excretory ducts of *G. urna* and tapeworms of the early diverging order Spathebothriidea (our unpublished data).

### New data on the fine morphology of the terminal organ of the basal cestode groups

Among the Neodermata, two types of the terminal protonephridial complex have been distinguished [13], one with septate junctions between terminal and adjacent duct cells (Monogenea and Trematoda), and the other without such septate junctions (Cestoda including Amphilinidea and Gyrocotylidea) [13, 25, 29, 35]. The present investigation revealed that the terminal organ in *G. urna* differs from those of all other tapeworms. In the places where the external ribs extend beyond the internal ones, the boundary between both cells is lined by electron-dense material forming contact sites (*zonulae adherentes*). Moreover, a septate junction is observed between terminal and the first duct cells at the level of the distal extremity of the flame tuft. It should be mentioned that the same structure of the terminal complex has been described for the monopisthocotylean *Anoplodiscus cirrusspiralis* and *Ancyrocephalus paradoxus* [37, 38]. Such terminal organ differs from those of other monogeneans as well as aspidogastreans and digeneans in the absence of a complete septate junction within the cytoplasmic cord and the absence of two cords on one side of the flame tuft. According to Rohde et al. [39], the complete junction has been secondarily lost in *A*. *cirrusspiralis*. The same pattern of the terminal complex in two monopisthocotyleans and gyrocotylidean cestode may be a characteristic supporting the existence of the clade Cercomeromorpha [3, 5]. It is also important to note that the protonephridial system of the Gyrocotylidea and both groups of the Monogenea have the same anatomy: two opening pores that are located in the anterior haft of the body [25]. In aspidogastrideans and digeneans there are two pores or a single pore in the posterior body and a single pore at the posterior end in post-larval Amphilinidea and Eucestoda (Nephroposticophora) [25]. Generally, the presence of septate junctions and lateral ciliary tufts in the excretory ducts of *G. urna* as well as the presence of the terminal complex with septate junction between two adjacent cells may support the basal position of gyrocotylideans among the tapeworms.

Surprisingly, the present study revealed a peculiar terminal cell with multiple ciliary tufts (*c.*5 µm long) with three to four cilia across the diameter of each bulb in an adult of *Amphilina foliacea*. The ciliary tufts of such a cell are distinguished by unique ultrastructural character of its weir, which consists of a single row of longitudinal dense ribs connected by a thin sheath of electron-dense material. In contrast, the typical type of the terminal flame cells of post-larval amphilinids was described to possess one tuft (*c*.8 µm long) with 15–20 cilia across its diameter and two rows of ribs [29]. Also, it is interesting to note that besides the presence of a ‘typical’ terminal cell with one ciliary tuft in the larva and adult of *Austramphilina elongata* [34, 39], a new cell type called ‘a multiciliated starcell’ was described in the larva of *A. elongata* [40]. It seems that the ‘multiciliated starcell’ in larval *A. elongata* belongs to the same morphological type as that in adult *A. foliacea*. Unfortunately, Rohde & Garlick [40] described this ‘multiciliated starcell’ based on a partially cut cell due to which they may have observed many mitochondria, scattered cilia and an intracellular cavity, into which leptotriches project from the cytoplasm. Despite a low number of cell sections, Rohde & Garlick [40] correctly estimated this ‘starcell’ as a new type of the terminal cell. Our present study demonstrates for the first time that such terminal cell of amphilinideans is a peculiar type of terminal cell, which is characterised by multiply ciliary tufts with their weir having a single row of ribs. It should be also noted that the terminal cells of free-living species of the Rhabdocoela and Lecithoepitheliata have a non-terminal perikaryon forming many ciliary tufts and possessing the ribs of the weir apparatus arranged in a single row [12, 13, 41]. Due to rare findings of such a terminal cell with multiple ciliary tufts in larval [40] and adult stages of amphilinideans (present study), we assume that such a protonephridial terminal cell may represent a rudimentary structure preserved in the amphilinidean phylogeny from the ancestor possessing such a peculiar terminal protonephridial cell type.

## Conclusions

The present investigation revealed the same pattern in the cytoarchitecture of the excretory ducts in species of the four major lineages of the Neodermata, which is similar to that present in proseriate and tricladid flatworms, i.e. the presence of septate junctions between adjacent epithelial cells and lateral ciliary tufts in the excretory ducts of the different levels. Therefore, the present ultrastructural study does not provide any support about close relationships of the basal neodermatans and *Bothrioplana semperi* because of different patterns of the ultrastructure of their excretory ducts. Kornakova [7] also assumed that the Neodermata derived from the ancestor belonging to an extinct group close to the Proseriata, but that scenario is not supported by molecular phylogenetic studies [1–3]. Several ultrastructural characters of gyrocotylideans such as the presence of the same structural pattern of the excretory ducts are shared with basal monopisthocotylidean and polyopisthocotylidean monogeneans, and aspidogastrean and digenean trematodes as well as with proseriates and tricladids. This supports the most basal position of the Gyrocotylidea among tapeworms. Among all tapeworms the unusual type of the protonephridia possessing a protonephridial cell forming many ciliary tufts with a single row of longitudinal ribs has been observed only in larval and adult amphilinideans ([40]; present study). Such unusual character preserved in the Amphilinidea, but absent in other tapeworms including more basal Gyrocotylidea corresponds to the observation by Waeschenbach et al. [42] that amphilinideans are highly divergent from eucestodes. In contrast, amphilinideans are characterised by syncytial structure of their excretory ducts, a character shared by all eucestodes, which supports the position of the Amphilinidea as the sister group to the Eucestoda [25, 42].

## Data Availability

All data supporting the conclusions of this article are included within the article.
